# Serological evaluation of coronavirus IgA and IgG antibodies in a repeated cross-sectional cohort of unvaccinated and vaccinated pregnant individuals over three months following SARS-CoV-2 infection

**DOI:** 10.1128/spectrum.02167-25

**Published:** 2025-12-05

**Authors:** Guadalein Tanunliong, Ana Citlali Márquez, Hind Sbihi, Tamara Pidduck, Yin Chang, Fang Fang Li, Danielle Luk, Lucia Forward, Elisabeth McClymont, Chelsea Elwood, Mel Krajden, Agatha N. Jassem, Deborah Money, Inna Sekirov

**Affiliations:** 1 Department of Pathology and Laboratory Medicine, University of British Columbia 8166, Vancouver, Canada; 2 Public Health Laboratory, British Columbia Centre for Disease Control, Vancouver, Canada; 3 Data and Analytics, British Columbia Centre for Disease Control, Vancouver, Canada; 4 School of Population and Public Health, University of British Columbia 8166, Vancouver, Canada; 5 Women’s Health Research Institute, Vancouver, Canada; 6 Department of Obstetrics and Gynecology, University of British Columbia 8166, Vancouver, Canada; Naturwissenschaftliches und Medizinisches Institut an der Universitat Tubingen, Reutlingen, Germany

**Keywords:** vaccines, COVID-19, endemic coronavirus, pregnancy, serology, SARS-CoV-2, antibodies

## Abstract

Antibody surveillance provided valuable public health insights during the Coronavirus Disease 2019 (COVID-19) pandemic. Severe Acute Respiratory Syndrome Coronavirus 2 (SARS-CoV-2) infection, vaccination, and cross-reactive responses from endemic coronaviruses (human coronaviruses [HCoVs]) can influence SARS-CoV-2 antibody responses, impacting reliability and interpretation of serological findings. Here, we investigated population-level SARS-CoV-2 and HCoV IgA and IgG responses following SARS-CoV-2 infection among unvaccinated and vaccinated pregnant individuals over 3 months. Using residual sera from routine antenatal screening of pregnant individuals in British Columbia between March 2020 to May 2022, we designed a retrospective repeated cross-sectional cohort of infected individuals either unvaccinated (UV + COV, *N* = 171) or two- to three-dose vaccinated (V + COV, *N* = 137). A total of 30 pre-pandemic sera served as negative controls. Sera were collected within 3 months of respiratory PCR-positivity in half-month intervals and tested for IgA and IgG against Spike (S) of alpha-HCoV (HCoV-229E, HCoV-NL63) and beta-HCoV (HCoV-HKU1, HCoV-OC43) and S, receptor binding domain, and nucleocapsid (N) of SARS-CoV-2 using a multiplex immunoassay. Following SARS-CoV-2 infection, V + COV had lower anti-N IgG levels (*P* = 0.004) and seropositivity rates than UV + COV. One month post-infection, V + COV (38%) had lower anti-N IgA seropositivity than UV + COV (73%). Both groups had significantly higher anti-S IgA and IgG levels against beta-HCoV vs controls, with signals correlating positively with SARS-CoV-2 anti-S levels for each isotype. These results suggest that neither IgA nor IgG can reliably identify recent infections in vaccinated populations, emphasizing the importance of considering complex interplay of antibody responses when interpreting serological data and recognizing the potential and limitations of serological testing for diagnostics and surveillance.

**IMPORTANCE:**

This study provides key insights into how Severe Acute Respiratory Syndrome Coronavirus 2 (SARS-CoV-2) vaccination and infection shape the magnitude and longevity of IgA and IgG antibody responses following infection in pregnant individuals. It also highlights the interplay of serological responses to related viruses, such as the human coronaviruses. By leveraging population-level antenatal sera, our findings highlight important considerations for the design and interpretation of future seroprevalence studies, antibody-based surveillance, and diagnostic strategies. As SARS-CoV-2 transitions into endemic circulation, understanding the complexity of SARS-CoV-2 antibody responses provides additional insights into the strengths and limitations of serological data interpretation in a real-world setting.

## INTRODUCTION

Surveillance of antibody responses (serosurveillance) generated from Severe Acute Respiratory Syndrome Coronavirus 2 (SARS-CoV-2) vaccination and infection provided valuable public health information during the Coronavirus Disease 2019 (COVID-19) pandemic (1). However, antibody responses across individuals exhibit significant heterogeneity due to varying infection, vaccination, and exposure histories, impacting the nature and breadth of serological findings. Understanding the antibody responses to SARS-CoV-2 and factors that influence these responses is crucial for accurate serological interpretations, which can help guide public health and vaccine policies (2).

Most SARS-CoV-2 serosurveillance studies rely on the detection of circulating immunoglobulin G (IgG) antibodies against the immunodominant regions of SARS-CoV-2, including the Spike (S) protein, the receptor binding domain (RBD) within the S region, and the nucleocapsid (N) protein, for estimates of infection and vaccination in the population (3, 4). Vaccinations in Canada have been S-based; thus, vaccinated individuals are expected to elicit antibody responses against S and RBD, permitting the detection of anti-N IgG as a marker for infection-induced antibody responses (3, 4). However, while SARS-CoV-2 infection following S-based vaccination typically results in higher antibody concentrations against the S protein compared to unvaccinated individuals, the opposite effect was observed for anti-N antibodies, with lower anti-N IgG seroconversion rates observed following SARS-CoV-2 infection among previously vaccinated individuals compared to those unvaccinated (5, 6). This prompts questions about the possible underestimation of infected individuals in vaccinated populations in serosurveillance studies (7), which must be further investigated, given that seroprevalence estimates obtained through these studies are used to guide vaccine booster policies.

The SARS-CoV-2 antibody response can also be influenced by cross-reactive antibodies to the highly genetically related endemic human coronaviruses (HCoVs) (8), including alpha-HCoV (HCoV-229E and HCoV-NL63) and beta-HCoV (HCoV-HKU1 and HCoV-OC43), the latter of which exhibits higher sequence similarities with SARS-CoV-2 (50%–51% sequence identity) (9). These four seasonal HCoVs have long been recognized to cause a substantial proportion of common colds (10, 11), with nearly everyone expected to develop and periodically boost immune memory to HCoVs through repeated re-exposures throughout their lifetime (11, 12). Cross-reactivity and back boosting of HCoV IgG antibodies have been previously demonstrated following SARS-CoV-2 infection, though their roles in either protection or exacerbation of infection remain inconsistent (13–18). Additionally, their potential influence in SARS-CoV-2 serology, whether serving as a supplemental serological marker or potentially hindering accurate detection of seroconversion due to the masking of a true SARS-CoV-2 response, needs to be further studied.

SARS-CoV-2’s transition into endemicity in a highly immunized population has overall brought to light several shortcomings of IgG as a serological marker of infection, including their inability to identify recent infections due to IgG’s persistence in circulation, potential underestimations of infection due to blunted anti-N responses following vaccinations, and the potential influence of cross-reactive HCoV responses. As such, there has been growing interest in expanding beyond IgG and using IgA as a potential marker to identify recent SARS-CoV-2 infection (19–21). While IgA is more commonly known in its dimeric and polymeric forms as a crucial player of mucosal defense, with mucosal IgA often associated with protection from viral replication and transmission at the local site of infection (22), systemic IgA can also exist in monomeric form in serum with a generally shorter half-life than IgG and has been variably associated with both disease severity and protection (23, 24). Both SARS-CoV-2 vaccination and infection generate IgA responses against S, RBD, and N (25, 26); however, the serum IgA dynamics over time following infection of previously vaccinated individuals is still unclear, and the cross-reactivity with HCoV at the IgA level, as well as serum IgA’s potential utility as a marker of recent infection, remains poorly understood.

Herein, we report on investigations of population-level IgA antibody dynamics to SARS-CoV-2 and HCoV, with respect to IgG, in a repeated cross-sectional cohort of vaccinated and unvaccinated pregnant individuals up to three months following SARS-CoV-2 infection. This work leveraged residual sera collected in British Columbia (BC), Canada, as part of routine antenatal communicable disease screening. Greater than 95% of all pregnant individuals in BC undergo antenatal screening during the first trimester, capturing diverse demographic and risk groups (27). This provides a relatively generalizable model to study SARS-CoV-2 antibody dynamics at a population level, given that trends of antibody waning remain consistent across populations. We hypothesized that IgA and IgG antibody responses to SARS-CoV-2 and HCoV vary according to the timing of serum collection following PCR-confirmed SARS-CoV-2 infection and the individual’s SARS-CoV-2 vaccination status pre-infection. As SARS-CoV-2 transitions into endemic circulation, our findings provide additional insights into the utility and limitations of serological testing for diagnostics and public health surveillance, which could help guide public health and vaccine policies.

## MATERIALS AND METHODS

### Study population

We used residual serum specimens collected during first trimester antenatal testing of pregnant individuals in BC, Canada, to design and implement a retrospective repeated cross-sectional study to investigate SARS-CoV-2 antibody responses to infection, vaccination, and previous HCoV antibodies. A schematic of our retrospective selection strategy is presented in Fig. S1. Sera were selected using linkages with BC’s integrated COVID-19 PCR testing data and COVID-19 vaccine registry to determine SARS-CoV-2 infection and vaccination histories and included individuals residing in all BC health regions, with the two most densely populated health regions notably over-represented in the selected cohort (Table 1). This study was approved by the Clinical Research Ethics Board of the University of British Columbia with a waiver of informed consent (H21-03566).

**TABLE 1:** Study population characteristics of individuals with documented PCR-positive SARS-CoV-2 infection (*N* = 308)^*a*^

	COVID-19 study population, *N* = 308		
Characteristics	UV + COV, *N* = 171	V + COV, *N* = 137	*P*-value
Age, median (IQR)	33.0 (30.0, 35.0)	32.0 (28.0, 36.0)	0.5^*b*^
Health Authority			0.009^*c*^
1	98 (57%)	56 (41%)	
2	18 (11%)	24 (18%)	
3	5 (2.9%)	5 (3.6%)	
4	40 (23%)	31 (23%)	
5	9 (5.3%)	20 (15)	
Unknown	1 (0.6%)	1 (0.7%)	
Sampling window	March 2020 – November 2021	August 2021 – April 2022	
SARS-CoV-2 PCR positive test to serum collection interval (median days and IQR)	58 (32, 77)	43 (28, 64)	0.009^*b*^
Time since last vaccination (median days and IQR)	NA^*d*^	142 (124)	
Vaccine doses pre-infection (N)			<0.001^*c*^
0	171 (100%)	0 (0%)	
2	0 (0%)	75 (55%)	
3	0 (0%)	62 (45%)	
Vaccine type: Dose 1	NA		
Vaxzevria		5 (3.6%)	
COVISHIELD		3 (2.2%)	
Spikevax		25 (18%)	
COMIRNATY		104 (76%)	
Vaccine type: Dose 2	NA		
Vaxzevria		1 (0.7%)	
Spikevax		37 (27%)	
CORMINATY		99 (72.3%)	
Vaccine type: Dose 3	NA		
Vaxzevria		37 (60%)	
Spikevax		25 (40%)	

^*a*^ Health authority refers to the specific regional organizations within the province responsible for managing and delivering public health services. UV + COV = unvaccinated prior to infection with SARS-CoV-2. V + COV = vaccinated with two or three doses prior to infection with SARS-CoV-2.

^*b*^ *P*-value from Wilcoxon rank sum test.

^*c*^ *P*-value from Chi-square test.

^*d*^ NA, not applicable.

A total of 338 serum specimens from 338 participants were included. Of these, 30/338 were collected prior to the index case in BC (28 January 2020) and served as pre-pandemic negative controls. A total of 308/338 sera were from individuals with a history of PCR-confirmed SARS-CoV-2 infection collected between March 2020 and May 2022. Of note, the provincial PCR testing strategy prior to December 2021 was to offer broad and accessible PCR testing to all BC residents, with a special emphasis on testing pregnant individuals. From December 2021 onward, PCR testing was only indicated for those requiring hospitalization or hospitalized, whereas rapid antigen test kits were largely distributed for community-based self-testing.

Among the 308 infected individuals, 44% (137/308) of the SARS-CoV-2 infected individuals were vaccinated prior to infection (V + COV group) with either two (2V + COV group, *N* = 75) or three (3V + COV group, *N* = 62) vaccine doses. The remaining 56% (171/308) of SARS-CoV-2 infected individuals were unimmunized, having no documented vaccine against SARS-CoV-2 on file prior to infection (UV + COV group). All vaccinations were Health Canada-approved at the time of administration (Vaxzevria, COVISHIELD, Spikevax, or COMIRNATY) and only expected to elicit an antibody response against the S and RBD proteins. All 308 sera were further grouped according to the timeframe between their SARS-CoV-2-positive PCR result and serum collection date at approximately half-month intervals, beginning at 0.5 months and ending at 3 months (Table S1). Individuals who had a documented positive SARS-CoV-2 PCR test prior to their vaccinations were excluded from the V + COV group of the study. See Table 1 for a detailed breakdown of demographic characteristics of all included participants.

### Detection of IgA and IgG antibody against ancestral SARS-CoV-2 and HCoV proteins using a multiplex electrochemiluminescent immunoassay

All residual sera (*N* = 338) were tested for antibodies against S, N, and S1 RBD of ancestral SARS-CoV-2, and the S proteins of four HCoVs (Alpha-HCoVs: HCoV-229E and HCoV-NL63, and Beta-HCoVs: HCoV-HKU1 and HCoV-OC43) using the quantitative multiplex V-PLEX Coronavirus Panel 2 Assay from Meso Scale Diagnostics (MSD, Rockville, USA), which was previously validated in-house for both IgG and IgA (Fig. S2). Assays were performed according to the manufacturer’s protocol as described previously (28, 29), briefly outlined below.

Ten-spot assay plates from MSD with antigens against S, RBD, and N regions of SARS-CoV-2, and S proteins of HCoV-229E, HCoV-NL63, HCoV-HKU1, and HCoV-OC43 printed on each spot were used for antibody testing. Assay plates were first blocked with MSD Blocker A for 30 minutes and then washed. Reference standard, assay controls, and sera (diluted 1:5,000 in MSD Diluent 100) were added and incubated for 2 hours before washing. Plates were then incubated with either the SULFO-TAG anti-human IgG or IgA detection antibody for 1 hour. Lastly, MSD Gold Read Buffer B was added to the plate following a final wash, and signals were immediately measured on the MSD QuickPlex SQ120. All incubation steps were carried out shaking at 700 rpm at room temperature. All wash steps were performed three times with MSD wash buffer prior to the addition of subsequent reagents outlined above.

Raw signals generated by the MSD QuickPlex SQ120 were processed using the MSD Discovery Workbench software (Version 4.0) and then imported into R (Version 3.6.2) and RStudio (Version 1.2.5033) to interpret signal cutoff values. IgG positivity threshold cutoffs for SARS-CoV-2 were provided by the manufacturer as follows: anti-SARS-CoV-2 S values above 1,960 AU/mL, anti-SARS-CoV-2 N values above 5,000 AU/mL, and anti-SARS-CoV-2 S1 RBD values above 538 AU/mL, and have been previously validated in our laboratory to demonstrate excellent sensitivity and specificity (28). Threshold cutoffs for IgA positivity for SARS-CoV-2 were previously developed and validated in-house using 30 pre-pandemic individuals and 51 acute COVID-19 patients and were as follows: anti-SARS-CoV-2 S values above 1,245 AU/mL, anti-SARS-CoV-2 N values above 1,250 AU/mL, and anti-SARS-CoV-2 S1 RBD values above 530 AU/mL (Fig. S2).

### Statistical analysis

All statistical analysis and data visualizations were conducted on R (Version 3.6.2) and RStudio (Version 1.2.5033). Processed data obtained from the MSD immunoassay was visualized using the *ggplot2* (Version 3.3.3) and *ggpubr* (Version 0.6.0) packages on RStudio. Reactivity cutoffs for IgA positivity were determined by taking the value two standard deviations above the geometric mean antibody level of the pre-pandemic negative individuals for each of S, RBD, and N. Sensitivity, specificity, positive predictive values, and negative predictive values were calculated using the *cutpointR* (Version 1.1.1) and *epiR* (Version 2.0.41) packages.

Kruskal-Wallis, Dunn’s *post hoc* multiple comparisons, chi-squared, Wilcoxon rank-sum, and Spearman’s correlation calculations were conducted using *ggpubr*, *gtsummary* (Version 1.5.1), and *stats* (Version 3.6.2) packages. Antibody trends were fitted using locally weighted regression (loess) between points on *ggpubr*. All antibody levels and reactivity cutoff values were log10 transformed for analysis.

## RESULTS

### Study population

Sera from 308 pregnant individuals were collected between March 2020 and May 2022. All 308 individuals had documented PCR-confirmed SARS-CoV-2 infections, classified as vaccinated (V + COV, *N* = 137) or unvaccinated (UV + COV, *N* = 171) for SARS-CoV-2 prior to infection, according to the provincial COVID-19 vaccine registry. Individuals were further grouped based on the timing of their serum collection relative to their PCR-confirmed SARS-CoV-2 infection date, in approximately half-month intervals. The median (interquartile range [IQR]) age between both groups does not differ significantly, at 33 years (30, 35) for the UV + COV group and 32 years (28, 36) for the V + COV group. The sampling windows were from March 2020 to November 2021 for UV + COV, and August 2021 to April 2022 for V + COV, with median (IQR) days from infection to serum collection at 58 days (32, 77) for UV + COV and 43 days (28, 64) for V + COV. Given that only PCR-confirmed cases were included, not all infections in the province may have been captured in this data set (Dataset S1). Furthermore, serum from 30 additional individuals prior to the index case in BC was included and served as pre-pandemic negative controls.

### Population IgG responses against S, RBD, and N of SARS-CoV-2 over 3 months following SARS-CoV-2 infection in vaccinated and unvaccinated pregnant individuals

**Fig 1:**
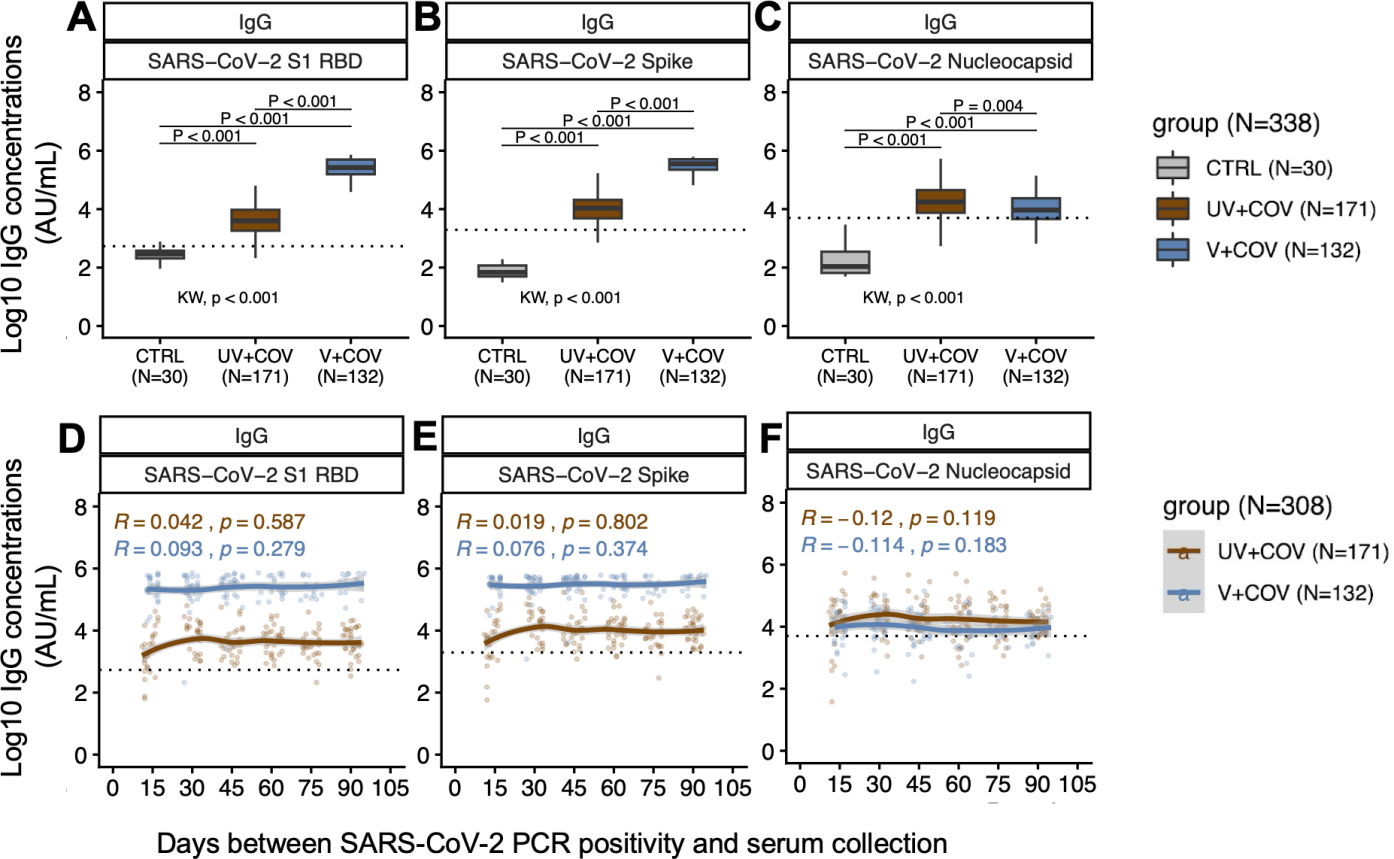
Population-level IgG antibodies against SARS-CoV-2 targets over 3 months in a repeated cross-sectional cohort of vaccinated (*N* = 132) or unvaccinated (*N* = 171) pregnant individuals prior to infection. Black dotted lines describe the respective positivity thresholds in log10 concentration (AU/mL) for each SARS-CoV-2 target. Alpha level for significance was set to *P* < 0.05. (**A through C**) Boxplots describe median IgG concentration and IQRs against SARS-CoV-2 RBD (**A**), S (**B**), and N (**C**) in the study population (*N* = 338). Antibody levels in UV + COV (brown, *N* = 171) and V + COV (blue, *N* = 132) groups were compared to pre-pandemic control individuals (gray, *N* = 30). Bonferroni-adjusted Kruskal-Wallis test was used to determine whether the three comparison groups were different, with *P*-values reported above the *x*-axis below the plots. *Post hoc* Bonferroni-adjusted Dunn’s test was used for pairwise multiple comparisons, with *P*-values presented alongside comparison bars at the top of each boxplot**.** (**D through F**) Each data point represents log10 antibody concentration (AU/mL) against SARS-CoV-2 RBD (**D**), S (**E**), and N (**F**) in sera collected from an individual in the UV + COV (brown) or V + COV (blue) groups. Non-parametric loess curves were plotted with shaded areas representing 95% confidence intervals. Spearman’s rank correlation coefficient rho (R) and corresponding *P*-values (*P*) were reported to describe the relationships between population antibody levels over time.

Following infection, V + COV individuals demonstrated significantly higher anti-S (*P* < 0.001) and anti-RBD IgG (*P* < 0.001) but lower anti-N IgG (*P* < 0.004), compared to UV + COV individuals (Fig. 1A through C). Anti-S and anti-RBD IgG remained consistently higher among V + COV across 3 months (Fig. S3), with stable IgG trends irrespective of two- or three-dose vaccinations (Fig. 1D and E; Fig. S4). IgG seropositivity rates remained high in V + COV serum over 3 months post-infection, ranging from 97% to 100% for both S and RBD (Table 2). Interestingly, among UV + COV individuals, IgG seropositivity rate was at 69%–100% for S and 77%–100% for RBD. Notably, the lower seropositivity rates at 69% for S and 77% for RBD among the UV + COV group were both observed during the 0.5-month time point. IgG seropositivity rates increased to 92%–100% for S and 96%–100% for RBD after 1 month post-infection (Table 2), consistent with the evolving seroconversion rates in the population following infection.

**TABLE 2:** SARS-CoV-2 anti-N, anti-RBD, and anti-S IgG seroprevalence in a repeated cross-sectional cohort of 308 pregnant individuals^*a*^.

IgG	UV + COV (*N* = 171)			V + COV (*N* = 137)		
	SARS-CoV-2 targets			SARS-CoV-2 targets		
Timing group (months)	N	RBD	S	N	RBD	S
0.5	21/26 (81%)	20/26 (77%)	18/26 (69%)	19/26 (73%)	26/26 (100%)	26/26 (100%)
1	23/26 (88%)	26/26 (100%)	26/26 (100%)	23/29 (79%)	28/29 (97%)	28/29 (97%)
1.5	24/27 (89%)	27/27 (100%)	26/27 (96%)	21/31 (68%)	31/31 (100%)	31/31 (100%)
2	25/32 (78%)	32/32 (100%)	30/32 (94%)	12/20 (60%)	20/20 (100%)	20/20 (100%)
2.5	20/26 (77%)	25/26 (96%)	24/26 (92%)	11/13 (85%)	13/13 (100%)	13/13 (100%)
3	31/34 (91%)	33/34 (97%)	34/34 (100%)	12/18 (67%)	18/18 (100%)	18/18 (100%)

^*a*^ UV + COV = unvaccinated prior to infection with SARS-CoV-2. V + COV = vaccinated with two or three doses prior to infection with SARS-CoV-2.

In contrast to anti-S and anti-RBD, we observed significantly lower anti-N IgG levels among V + COV compared to UV + COV individuals (*P* = 0.004) (Fig. 1C). When compared at each time point, V + COV individuals consistently exhibited lower median anti-N levels than UV + COV individuals, though these trends were not statistically significant (Fig. S3). Median antibody levels for anti-N remained stable over 3 months (Fig. 1F) for both UV + COV and V + COV, irrespective of two- or three-dose vaccinations (Fig. S4). Anti-N IgG seropositivity rates were lower at 60%–85% for V + COV, compared to 77%–91% for UV + COV (Table 2).

### Population IgA responses against S, RBD, and N of SARS-CoV-2 over 3 months following SARS-CoV-2 infection in vaccinated and unvaccinated pregnant individuals

**Fig 2:**
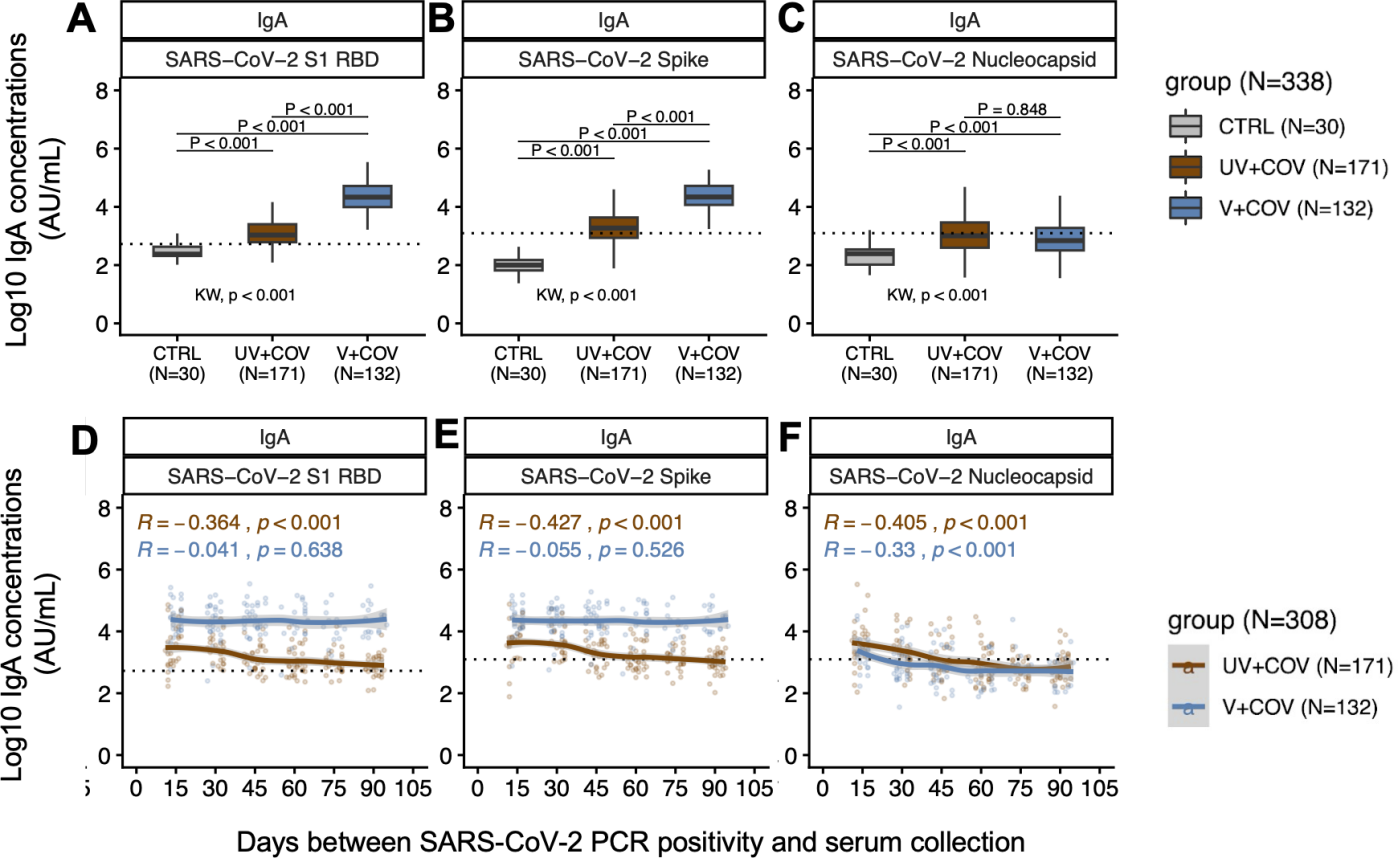
Population-level IgA antibodies against SARS-CoV-2 targets over 3 months in a repeated cross-sectional cohort of vaccinated (*N* = 132) or unvaccinated (*N* = 171) pregnant individuals prior to infection. Black dotted lines describe the respective positivity thresholds in log10 concentration (AU/mL) for each SARS-CoV-2 target. Alpha level for significance was set to *P* < 0.05. (**A through C**) Boxplots describe median IgA concentration and IQRs against SARS-CoV-2 RBD (**A**), S (**B**), and N (**C**) in the study population (*N* = 338). Antibody levels in UV + COV (brown, *N* = 171) and V + COV (blue, *N* = 132) groups were compared to pre-pandemic control individuals (gray, *N* = 30). Bonferroni-adjusted Kruskal-Wallis test was used to determine whether the three comparison groups were different, with *P*-values reported above the *x*-axis below the plots. *Post hoc* Bonferroni-adjusted Dunn’s test was used for pairwise multiple comparisons, with *P*-values presented alongside comparison bars at the top of each boxplot**.** (**D through F**) Each data point represents log10 antibody concentration (AU/mL) against SARS-CoV-2 RBD (**D**), S (**E**), and N (**F**) in sera collected from an individual in the UV + COV (brown) or V + COV (blue) groups. Non-parametric loess curves were plotted with shaded areas representing 95% confidence intervals. Spearman’s rank correlation coefficient rho (R) and corresponding *P*-values (*P*) were reported to describe the relationships between population antibody levels over time.

We observed significantly higher anti-S (*P* < 0.001) and anti-RBD (*P* < 0.001) IgA (Fig. 2A and B), but not anti-N IgA (Fig. 2C), following SARS-CoV-2 infection among V + COV compared to UV + COV individuals (Fig. S5). Anti-S and anti-RBD IgA trends appeared stable over 3 months for the V + COV group (Fig. 2D and E), irrespective of two- or three-dose vaccinations (Fig. S4), but declined over time in the UV + COV group, as demonstrated by the loess curves (Fig. 2D and E). The V + COV group exhibited higher IgA seropositivity rates at 95%–100% for both S and RBD for each timing group, in contrast to the lower UV + COV IgA seropositivity rates at 44%–85% for S and 75%–100% for RBD (Table 3). Notably, anti-S IgA seropositivity rates for the UV + COV group for the 0.5 and 1-month post-infection groups were both at 85%, with decreases in the seropositivity rates seen as the timeframe increases, reaching 44% by 3 months post-infection (Table 3).

**TABLE 3:** SARS-CoV-2 anti-N, anti-RBD, and anti-S IgA seroprevalence in a repeated cross-sectional cohort of 308 pregnant individuals^*a*^

IgA	UV + COV (*N* = 171)			V + COV (*N* = 137)		
	SARS-CoV-2 targets			SARS-CoV-2 targets		
Timing group (months)	N	RBD	S	N	RBD	S
0.5	21/26 (81%)	23/26 (88%)	22/26 (85%)	19/26 (73%)	26/26 (100%)	25/26 (96%)
1	19/26 (73%)	26/26 (100%)	22/26 (85%)	11/29 (38%)	28/29 (97%)	28/29 (97%)
1.5	10/27 (37%)	22/27 (81%)	18/27 (67%)	10/31 (32%)	31/31 (100%)	30/31 (97%)
2	11/32 (34%)	24/32 (75%)	15/32 (47%)	3/20 (15%)	19/19 (95%)	19/20 (95%)
2.5	5/26 (19%)	21/26 (81%)	13/26 (50%)	3/13 (23%)	13/13 (100%)	13/13 (100%)
3	9/34 (26%)	26/34 (76%)	15/34 (44%)	3/18 (17%)	18/18 (100%)	18/18 (100%)

^*a*^ UV + COV = unvaccinated prior to infection with SARS-CoV-2. V + COV = vaccinated with two or three doses prior to infection with SARS-CoV-2.

V + COV individuals exhibited lower anti-N IgA concentrations relative to UV + COV individuals, but this difference was not statistically significant (*P* = 0.848) (Fig. 2F; Fig. S5). Anti-N IgA seropositivity rates declined over 3 months for both UV + COV and V + COV (Table 3), though this decline occurred earlier for the V + COV group (73% at 0.5 months to below 38% at 1 month onward) compared to the UV + COV group (81% at 0.5 months, to 73% at 1 month, to below 37% at 1 month onward) (Table 3).

### Cross-reactive HCoV IgA and IgG responses following SARS-CoV-2 infection in vaccinated and unvaccinated pregnant individuals

**Fig 3:**
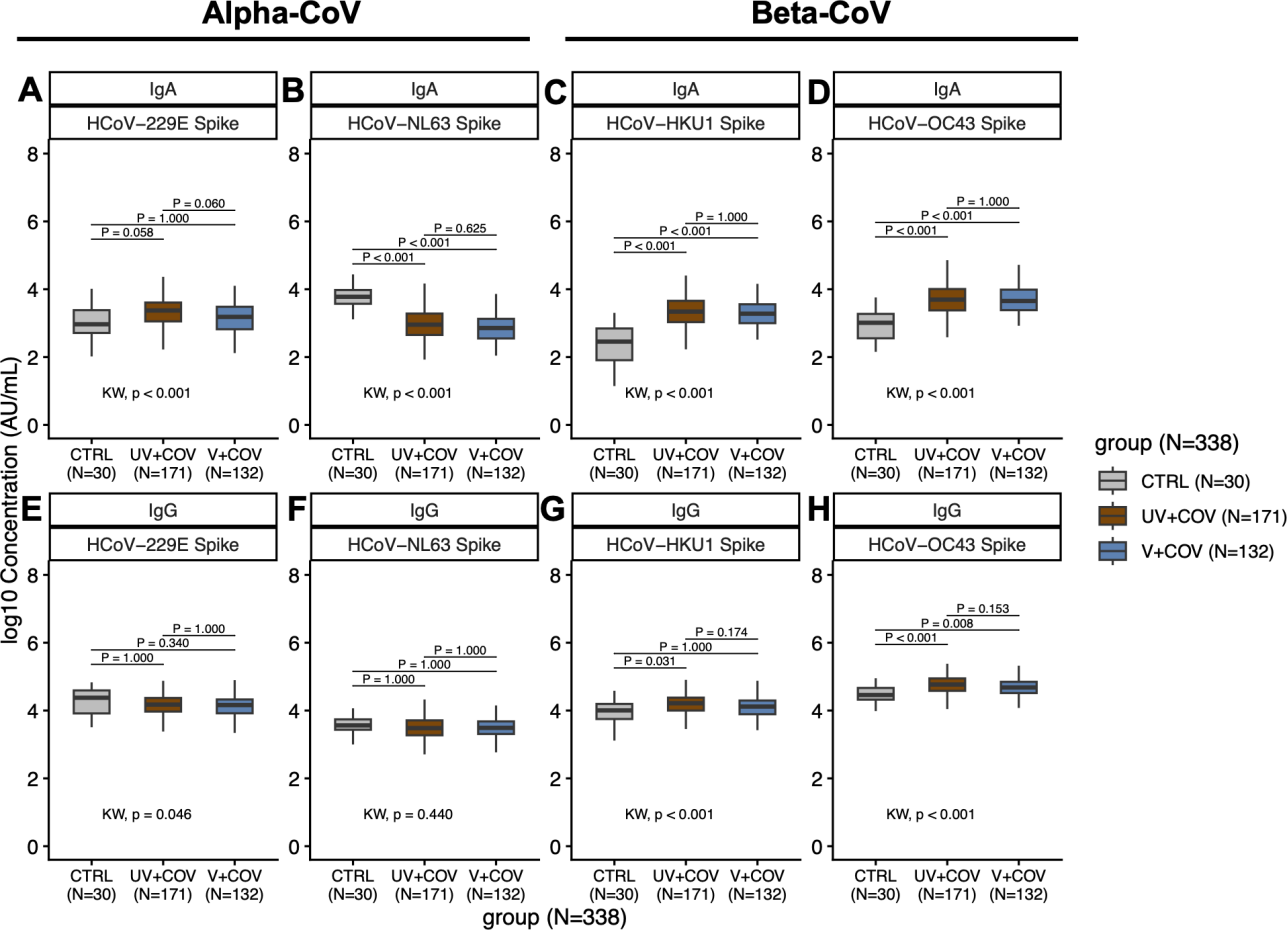
IgA (**A through D**) and IgG (**E through H**) antibody concentration against HCoV S in SARS-CoV-2 infected pregnant individuals (*N* = 308) compared to pre-pandemic pregnant controls (CTRL, *N* = 30)**.** Boxplots describe median concentration and IQRs of IgA (**A through D**) or IgG (**E through H**) antibodies against the S region of alpha-HCoVs HCoV-229E (**A and E**) and HCoV-NL63 (**B and F**) as well as beta-HCoVs HCoV-HKU1 (**C and G**) and HCoV-OC43 (**D and H**). Antibody levels in UV + COV (brown, *N* = 171) and V + COV (blue, *N* = 132) groups are compared to pre-pandemic control individuals (gray, *N* = 30). Black dotted lines describe the respective reactivity thresholds in log10 concentration (AU/mL) for SARS-CoV-2 targets. Bonferroni-adjusted Kruskal-Wallis test was used to determine whether the three comparison groups are different, with *P*-values reported above the *x*-axis below the plots. *Post hoc* Bonferroni-adjusted Dunn’s test was used for pairwise multiple comparisons, with *P*-values presented alongside comparison bars at the top of each boxplot. Alpha level for significance was set to *P* < 0.05.

When compared to pre-pandemic controls, both UV + COV and V + COV individuals exhibited significantly higher IgA levels against beta-HCoVs HCoV-HKU1 (*P* < 0.001) and HCoV-OC43 (*P* < 0.001), but not alpha-HCoVs (Fig. 3A through D). Similarly, for IgG, compared to pre-pandemic controls, anti-OC43 IgG levels were higher among UV + COV (*P* < 0.001) and V + COV (*P* = 0.008), and anti-HKU1 IgG levels were higher among the UV + COV group (*P* = 0.031), while no significant differences were observed for alpha-HCoV IgG (Fig. 3E through H). Notably, anti-NL63 IgA, but not IgG, was lower following SARS-CoV-2 infection for both UV + COV (*P* < 0.001) and V + COV (*P* < 0.001) relative to levels observed in sera collected pre-pandemic (Fig. 3B and F).

**Fig 4:**
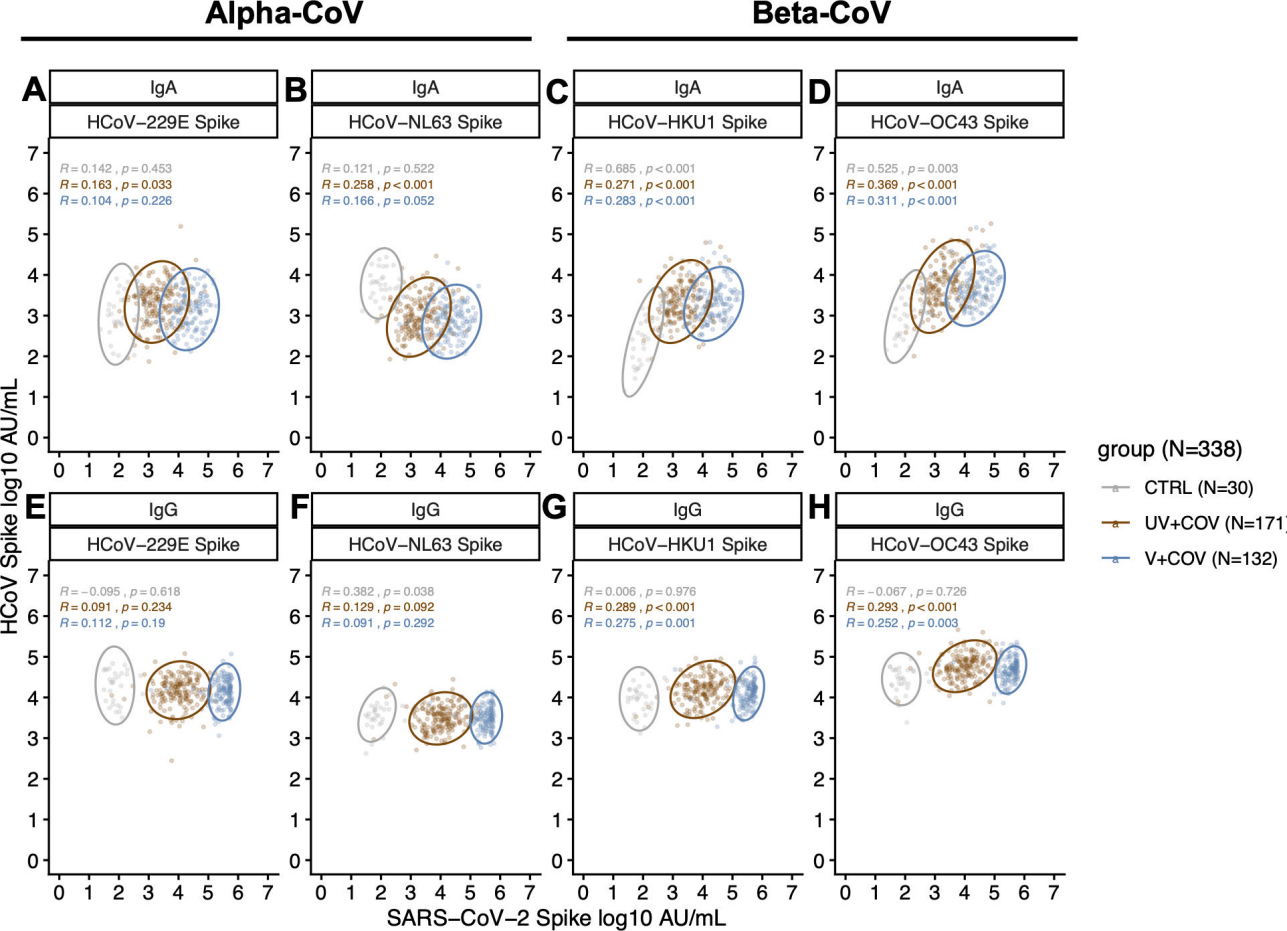
IgA (**A through D**) and IgG (**E through H**) antibody concentration against HCoV S in SARS-CoV-2 infected pregnant individuals (*N* = 308) compared to pre-pandemic pregnant controls (CTRL, *N* = 30)**.** Scatterplot of IgA (**A through D**) or IgG (**E through H**) antibodies against the S region of alpha-HCoVs HCoV-229E (**A and E**) and HCoV-NL63 (**B and F**) as well as beta-HCoVs HCoV-HKU1 (**C and G**) and HCoV-OC43 (**D and H**) correlated with SARS-CoV-2 S antibody levels. Circles represent 95% ellipses. Data points in UV + COV (brown, *N* = 171) and V + COV (blue, *N* = 132) groups are compared to pre-pandemic control individuals (gray, *N* = 30). Spearman’s correlation coefficient (Rho, R) and corresponding *P*-values are presented.

Correlation analysis demonstrates that compared to infected individuals, pre-pandemic controls exhibited stronger positive correlations between SARS-CoV-2 S IgA and beta-HCoV S IgA levels (HCoV-HKU1: Rho = 0.685, *P* < 0.001; HCoV-OC43: Rho = 0.525, *P* = 0.003), but not for alpha-HCoV (Fig. 4A through D), suggesting pre-existing cross-reactivity at the IgA level for beta-HCoV (Fig. 4C and D). Infected individuals exhibited mildly significantly positive correlations between SARS-CoV-2 S and beta-HCoV S levels for both IgA and IgG, but not alpha-HCoV (Fig. 4E through H). In contrast to IgA, pre-pandemic SARS-CoV-2 S IgG and beta-HCoV S IgG were not significantly correlated (Fig. 4G and H). Notably, SARS-CoV-2 S IgA was significantly positively correlated with anti-NL63 IgA (R = 0.258, *P* < 0.001) for the UV + COV group (Fig. 4B).

## DISCUSSION

The COVID-19 pandemic highlighted the value of serological testing for public health surveillance and guiding vaccine policy, underlining the importance of understanding antibody responses in SARS-CoV-2 infection. Here, we utilized residual sera collected from routine antenatal screening in BC to investigate the SARS-CoV-2 and HCoV IgA and IgG antibody responses in vaccinated and unvaccinated pregnant individuals over 3 months following SARS-CoV-2 infection.

We observed that following infection, vaccinated individuals had higher and more stable levels of anti-S and anti-RBD IgG but significantly lower levels of anti-N IgG, compared to those unvaccinated. The lower levels of anti-N IgG and lower rates of anti-N seropositivity among vaccinated individuals were consistent with other studies showing anti-N blunting and lower seroconversion rates following SARS-CoV-2 infection of previously vaccinated individuals (5–7, 30). When compared by timing groups, while vaccinated individuals consistently demonstrated lower anti-N IgG trends compared to those unvaccinated, this difference did not reach statistical significance, which could be attributed to the relatively small sample size. Nonetheless, this consistent trend of blunted anti-N IgG has been attributed to a rapid and stronger recall of anamnestic anti-S responses following infection of vaccinated individuals, leading to limited viral entry, limited viral replication, faster viral clearance, and an overall lower induction of anti-N antibodies (7, 30). As such, anti-N IgG responses may be a less reliable serological indicator of past infection in vaccinated populations, and potentially in previously infected populations. Given that many serological assay validations were designed and evaluated in unvaccinated populations prior to the deployment of vaccines, it is imperative that assay performance be assessed in a real-world setting, particularly in vaccinated populations, to prevent underestimation of infected individuals in seroprevalence studies. With the rapid emergence of variants that continue to change the SARS-CoV-2 immune landscape, infections and vaccinations may induce differentially boosted binding antibodies and neutralization responses across variants (31, 32). This highlights the importance of ongoing investigations of both the SARS-CoV-2 serological landscape and the performance of serological assays to ensure accurate serological interpretations for diagnostics and surveillance. Furthermore, it may also be necessary to adjust assay cutoffs for serological positivity given vaccination status and/or undertake repetitive sampling to infer seroprevalence more reliably over shorter time frames, rather than consider that seroprevalence estimates hold over long periods of time.

IgG has been commonly used as a serological marker to identify past infection and/or vaccination due to their systemic persistence, which can be attributed to both the ongoing systemic IgG production by memory B cells and IgG’s own recycling mechanism (33). Specifically, IgG’s interactions with the neonatal fragmental crystallizable receptor (FcRn) promote its protection from intracellular lysosomal degradation (33), recycling IgG back into circulation, and supporting its serological use for identification of past infection and/or vaccination. In contrast, IgA’s shorter half-life can be attributed in part to its lack of protection from degradation due to a lack of this FcRn region (34, 35), suggesting that high levels of circulating IgA may potentially be correlated with recent infections and/or vaccination. In our study, we found that unvaccinated individuals demonstrated primary IgA responses to S, RBD, and N that declined below seropositivity cutoffs within 3 months post-infection, suggesting that higher population IgA levels may indeed be better correlated with recent infection among unvaccinated populations. However, given that vaccinated individuals exhibited stable levels of anti-S and anti-RBD IgA over 3 months following infection, IgA’s potential use in identifying recent infections may not be applicable in highly vaccinated settings. While IgA’s short-lived nature had consistently gathered interest for its potential utility in identifying recently infected individuals, given the large heterogeneity in population seropositivity rates and the stable IgA recall responses observed among vaccinated individuals, individual IgA antibody levels are not likely to be a reliable marker of recent infections, especially within a recently vaccinated population.

SARS-CoV-2’s transition into endemicity prompts the need for an improved understanding of its serological response dynamics and its interactions with the immune response to other HCoVs to better understand non-specific and cross-reactive responses across coronaviruses. We observed a weakly positive correlation between beta-HCoV anti-S IgG and SARS-CoV-2 anti-S IgG following infection, irrespective of prior vaccination status. This correlation was absent in pre-pandemic individuals, suggesting either antibody cross-reactivity or a boosting of specific beta-HCoV IgG antibodies upon SARS-CoV-2 infection. Our findings were consistent with other studies that have described an induction of beta-HCoV memory B cells upon SARS-CoV-2 infection (17, 36, 37), specifically against the conserved S2 region within the S protein (8, 38), suggesting induction of beta-HCoV memory responses beyond antibody cross-reactivity in circulation. Furthermore, several studies have evaluated the association between cross-reactive beta-HCoV responses with COVID-19 severity (8, 15, 39), though their role in protection from severe COVID-19 remains uncertain. Similar to IgG, we observed that infected individuals had higher beta-HCoV IgA compared to pre-pandemic individuals, and these beta-HCoV IgA were positively correlated with SARS-CoV-2 anti-S IgA, though weaker than the correlations observed in pre-pandemic control individuals. This could indicate higher baseline cross-reactivity for beta-HCoV IgA, relative to IgG, and the decrease in IgA cross-reactivity following infection could be due to a relatively higher proportion of SARS-CoV-2-specific S1 (rather than the conserved S2) IgA responses post-SARS-CoV-2 infection. Of note, we also observed lower levels of the alpha-HCoV anti-NL63 IgA, but not IgG, among SARS-CoV-2-infected individuals when compared to pre-pandemic sera. While unexpected, the decrease in IgA, but not IgG, may be due to lower overall circulation of respiratory viruses in BC when COVID-19 pandemic measures had been in place, and the decline in IgA, but not IgG, may reflect the more rapid waning kinetics of IgA relative to IgG (40). To date, we are one of the few studies to demonstrate increased HCoV IgA responses following acute infection in this specific population (41, 42). Future studies should consider if this reflects cross-reactive induction of IgA by conserved epitopes or boosting of HCoV-specific memory responses and whether this confers cross-protective effects against infection and disease. While we observed an increase in beta-HCoV IgA and IgG following SARS-CoV-2 infection, similar to findings in early pandemic studies, we are unable to discern whether this is due to cross-reactivity or specific boosting of beta-HCoV responses. Several studies have suggested an association of elevated beta-HCoV responses with either disease severity or protection from infections and severe disease. While Guo et al. (43) observed an association with disease severity where higher HCoV-OC43 S IgG levels were associated with patients with more severe disease, Ortega et al. (44) demonstrated an association with protection, where higher trends of anti-HCoV N IgA and IgG were observed among asymptomatic compared to symptomatic participants following SARS-CoV-2 infection. In contrast, Anderson et al. (45) demonstrated that while beta-HCoV antibodies were boosted following infection with SARS-CoV-2, these were not associated with disease severity or protection. The heterogeneity in these findings could be partially attributed to the variability in methodologies, such as testing of antibodies against different HCoV targets, the use of various assays of distinct sensitivities and specificities (46), and other variations in population characteristics and exposure histories. Further studies are needed to investigate these cross-reactive HCoV responses and their influences on the SARS-CoV-2 antibody responses and COVID-19 outcomes.

Our study has several limitations, including the small sample size and the lack of paired longitudinal samples from the same individual over time, hindering antibody level comparisons at an individual level. Nonetheless, our use of repeated cross-sectional sampling produces estimates of aggregate population-level changes over time by use of a relatively unbiased representative sample at every time point, improving its external validity. Additionally, we acknowledge that there was a substantial underestimation of infectious case-based surveillance reporting described during the Omicron period in 2022 (4, 27), as the broadly accessible PCR testing strategy in BC had changed from December 2021 onward to being indicated only for those hospitalized; thus, by using PCR as a criterion for infection, we likely missed asymptomatic or mildly symptomatic infections and individuals who chose not to seek testing during this time. Nevertheless, our study did not intend to estimate BC’s overall infection prevalence and vaccine coverage; but rather, we aimed to design a repeated cross-sectional study to specifically compare the changes in population-level antibody responses to SARS-CoV-2 infection over time among previously vaccinated vs unvaccinated individuals.

We also acknowledge that another limitation of our study is the wide sampling timeframe, with the V + COV group mostly sampled at a later date than UV + COV. As our assay was designed for ancestral SARS-CoV-2, antibody responses to later variants may exhibit reduced binding affinity and therefore be underestimated; however, there remains substantial cross-reactivity in antibody responses across conserved epitopes, including N; thus, this is unlikely to have a substantial impact on our conclusions. Furthermore, despite the timeframe to serum collection being statistically significantly different between the UV + COV and V + COV groups at a median (IQR) of 58 days (32, 77) and 43 days (28, 64), respectively, the large overlap in spread between both groups suggests that this statistical difference is unlikely to translate into biological differences. Specifically, antibody levels have been shown to peak and plateau within the sampling timeframe assessed in this study; thus, this difference is unlikely to bias our findings. In addition, differences in timing between previous vaccination prior to subsequent infections could contribute to some variability in measured antibody levels among vaccinated individuals, reflecting real-world population heterogeneity.

Finally, our study uses a pregnant population, which includes individuals of childbearing age. Clinical and demographic characteristics such as smoking status, disease severity, underlying comorbidities, and co-medications could not be collected for this cohort within the parameters of the study design and therefore could not be accounted for in the analysis. Nonetheless, while our findings are not directly generalizable to other age groups, such as children, older adults, males, and more vulnerable populations, antibody waning occurs in all these populations, so the trends are relatively generalizable as well. Additionally, in BC, prenatal screening has an exceptional uptake of >95% and represents individuals from a wide range of demographic characteristics, geographic jurisdictions, and socioeconomic and cultural backgrounds (27). It is also a cohort of generally healthy individuals, in contrast to many other types of residual sera collected clinically that might be biased toward individuals with underlying health conditions. Thus, our cohort presents a unique and less biased sample set relative to hospitalized patients for seroprevalence studies utilizing residual clinical specimens (27).

A major study strength lies in the use of a highly sensitive assay for simultaneous detection of SARS-CoV-2 and HCoV antibodies (28), minimizing biases that might arise due to variable specimen handling during the testing process. Another strength is the use of the provincial public health laboratory SARS-CoV-2 PCR testing database and the provincial COVID-19 vaccine registry to classify individuals according to SARS-CoV-2 PCR testing results and vaccination status, minimizing misclassification biases that could arise from self-reported results.

Overall, we found differences in IgA and IgG antibody levels against S, RBD, and N following SARS-CoV-2 infection, which was associated with both vaccination history and the timing of serum collection relative to infection. We also observed higher beta-HCoV HKU1 and OC43 IgA and IgG levels following confirmed SARS-CoV-2 infection relative to pre-pandemic levels. Our findings highlight the importance of considering these factors and the continuous re-evaluations of the coronavirus serological landscape to enable more reliable serological interpretations and guide clinical, public health, and vaccine policies.

## Data Availability

All data in the article and supplemental material are available upon request from the authors.

## SUPPLEMENTAL MATERIAL

Supplemental material spectrum.02167-25-s0001.docx Figures S1 to S5; Table S1.

Dataset S1 spectrum.02167-25-s0002.csv Serology results for samples used in the study.

ASM does not own the copyrights to Supplemental Material that may be linked to, or accessed through, an article. The authors have granted ASM a non-exclusive, world-wide license to publish the Supplemental Material files. Please contact the corresponding author directly for reuse.
