# Supplementary material for: Serological evaluation of coronavirus IgA and IgG antibodies in a repeated cross-sectional cohort of unvaccinated and vaccinated pregnant individuals over three months following SARS-CoV-2 infection
Source: Microbiol Spectr. 2025 Dec 5;14(1):e02167-25. doi: 10.1128/spectrum.02167-25 (PMC12772236; doi:10.1128/spectrum.02167-25)
Supplement: Supplemental material — Figures S1 to S5; Table S1. [file spectrum.02167-25-s0001.docx]

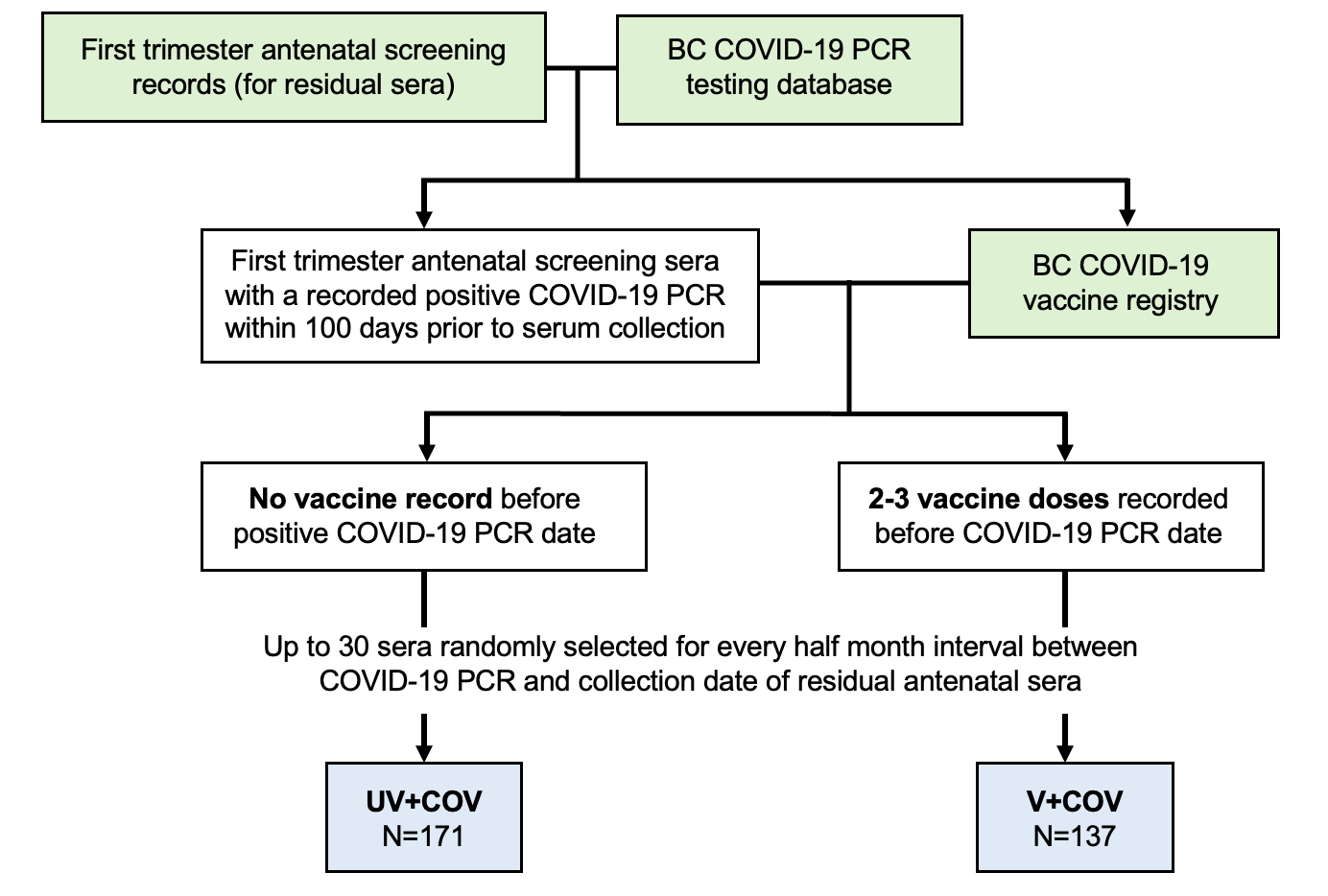


**Supplementary Figure 1. Schematic of the retrospective selection strategy of study population.** Green boxes indicate British Columbia’s (BC) provincial databases. White boxes indicate selection criteria following linkage of databases. Blue boxes represent the study population. First trimester antenatal screening records for residual sera were linked to BC’s COVID-19 PCR testing database to select residual sera with a positive COVID-19 PCR record within approximately 3 months prior to serum collection. These were linked to the BC COVID-19 vaccine registry for further stratification, and individuals with either no COVID-19 vaccine documented (UV+COV group) or 2-3 COVID-19 vaccine doses recorded (V+COV group) before PCR testing were selected. Up to 30 individuals were randomly selected at each half month interval between PCR testing and serum collection for each of the UV+COV and V+COV groups (See Supplementary Table 1).


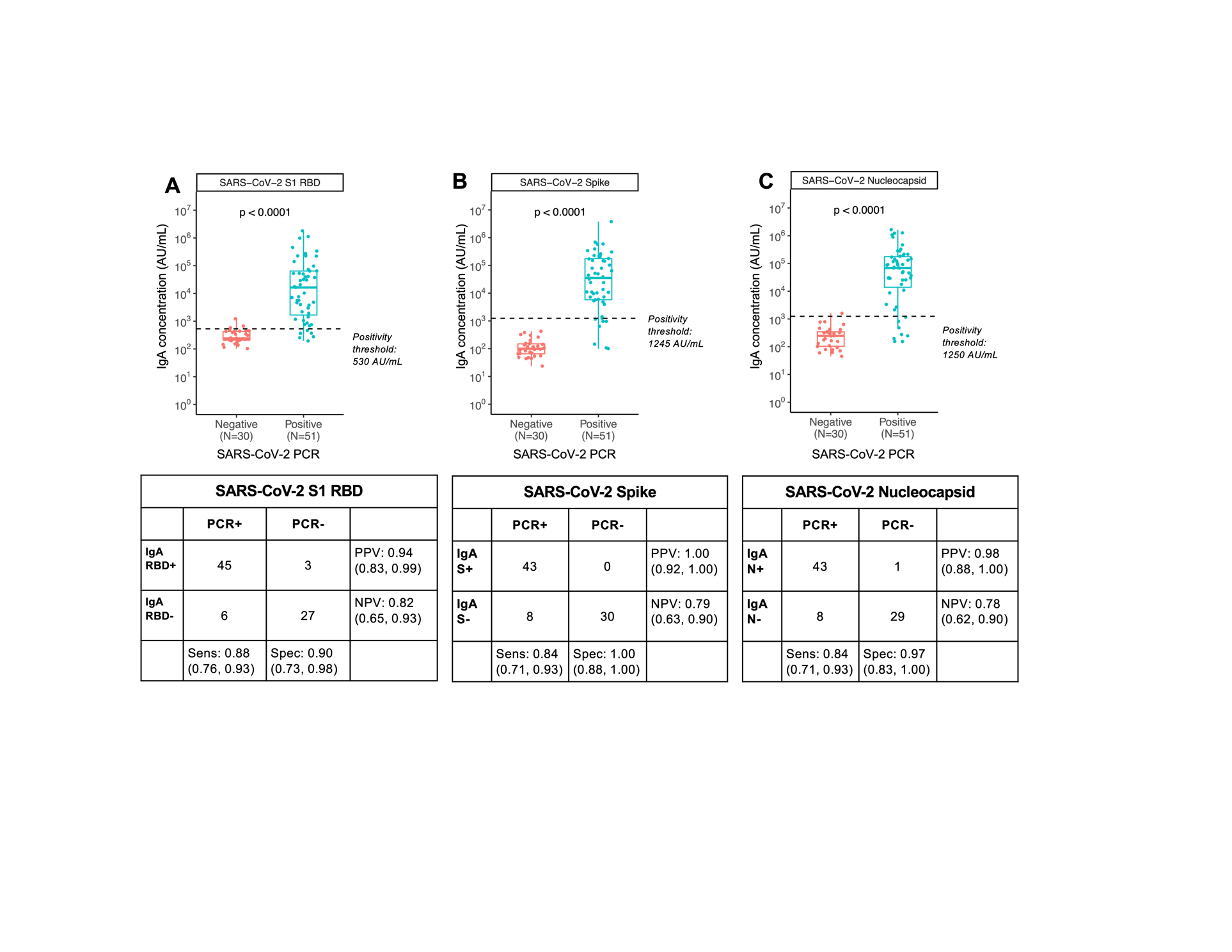


**Supplementary Figure 2. Validation of assay positivity cutoffs for detection of IgA antibody responses targeting SARS-CoV-2 RBD, S, and N using a validation cohort of 30 pre-pandemic negatives and 51 SARS-CoV-2 PCR positive cases.** Specimens were derived from previous validations ^25,26^. IgA antibody levels for RBD (A), S (B), and N (C) are shown in boxplots for both negative controls (red) and PCR positive cases (blue). Dashed lines correspond to positivity threshold for each target. P-value was obtained from Bonferroni-adjusted Wilcoxon rank-sum test, with α=0.05. A contingency table, assay sensitivity (Sens), specificity (Spec), positive predictive value (PPV), and negative predictive value (NPV) for each antibody target is described below each respective figure.


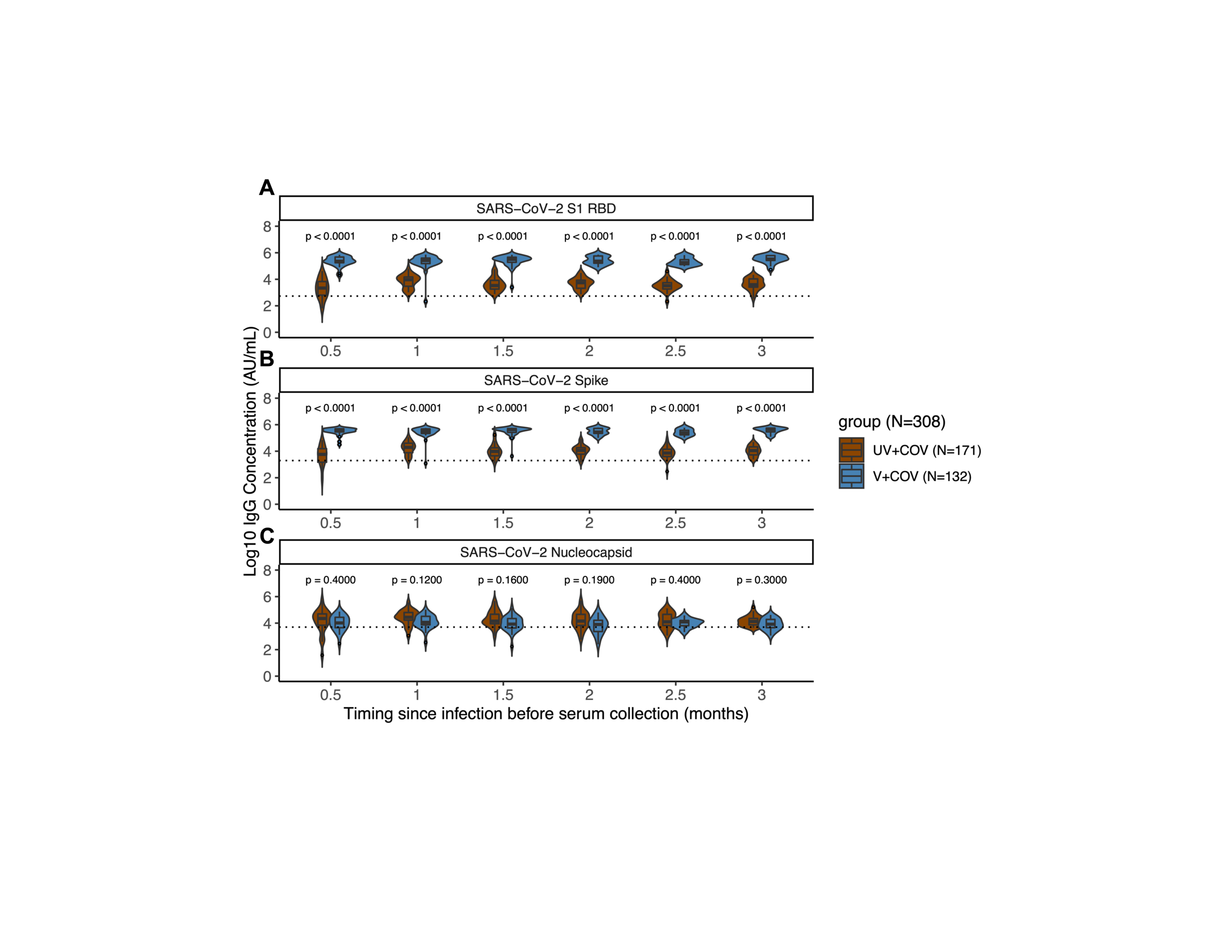


**Supplementary Figure 3. Violin and box plots for comparisons of median IgG levels between vaccinated and unvaccinated pregnant individuals at each timepoint for SARS-CoV-2 RBD (A), S (B), and N (C).** Boxplots describe median concentration and interquartile ranges. Antibody levels in UV+COV (brown) are compared to V+COV (blue) groups. Black dotted lines describe the respective reactivity thresholds in log10 concentration (AU/mL) for SARS-CoV-2 targets. Bonferroni-adjusted Wilcoxon rank-sum test was used to determine whether the two comparison groups are different at each timepoint, with P-values reported above each comparison. Alpha level for significance was set to P<0.05.


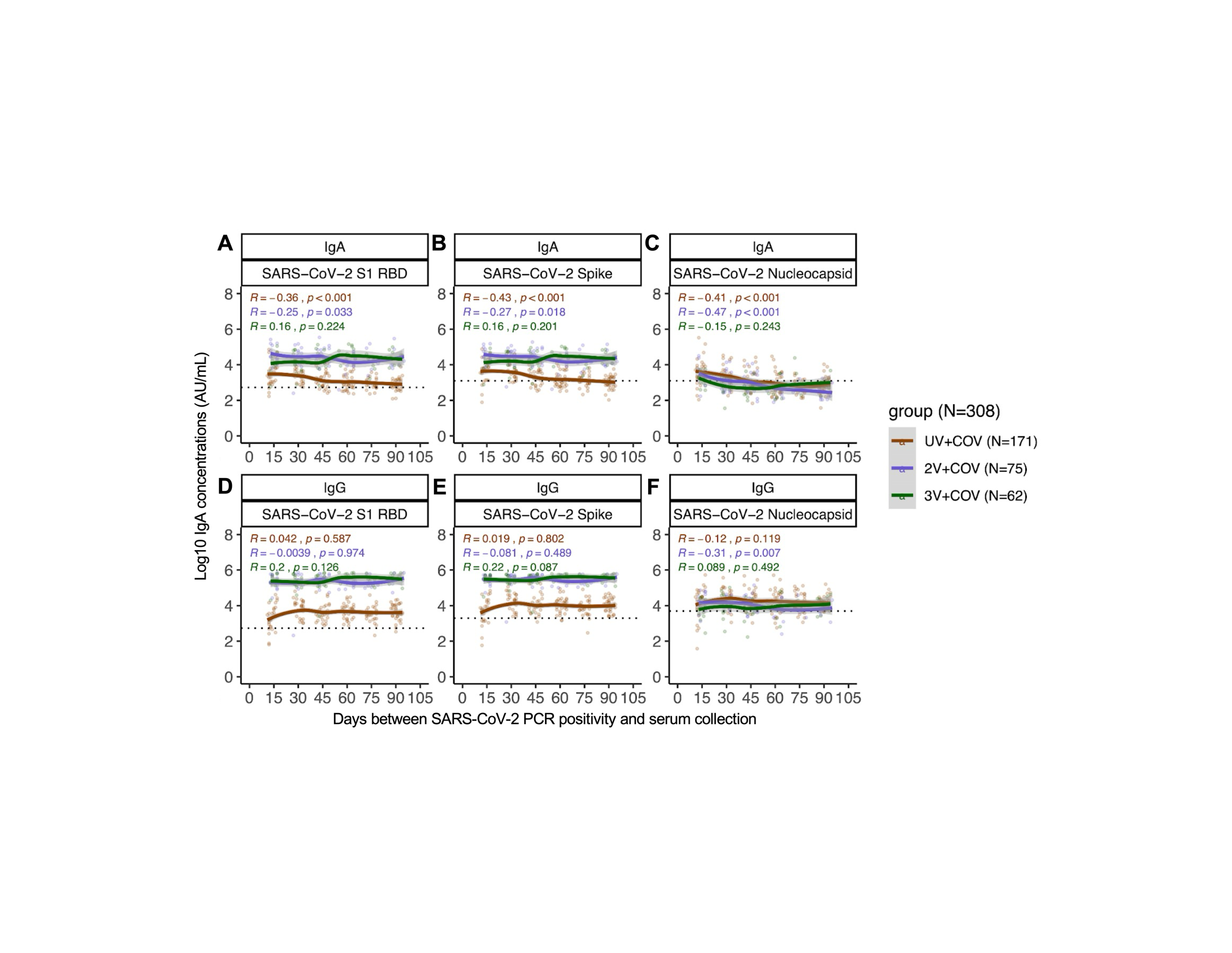


**Supplementary Figure 4.** **Population-level changes in IgA *(A-C)* and IgG *(D-F)* antibodies against SARS-CoV-2 targets over three months in a repeated cross-sectional cohort of pregnant individuals (N=308) 2-dose vaccinated (N=75), 3-dose vaccinated (N=62), or unvaccinated (N=171) prior to infection.** Each data point represents log10 antibody concentration (AU/mL) against SARS-CoV-2 RBD *(A, D)*, S *(B, E)*, or N *(C, F)* in sera collected from a single individual. Non-parametric locally weighted regression (LOESS) curves were plotted with shaded areas representing 95% confidence intervals. Spearman’s rank correlation coefficient rho (R) and corresponding P-values (P) were reported to describe the relationships between population antibody levels over time. Black dotted lines describe the respective positivity thresholds in log10 concentration (AU/mL) for SARS-CoV-2 targets in IgA and IgG immunoassays. Alpha level for significance was set to P<0.05.


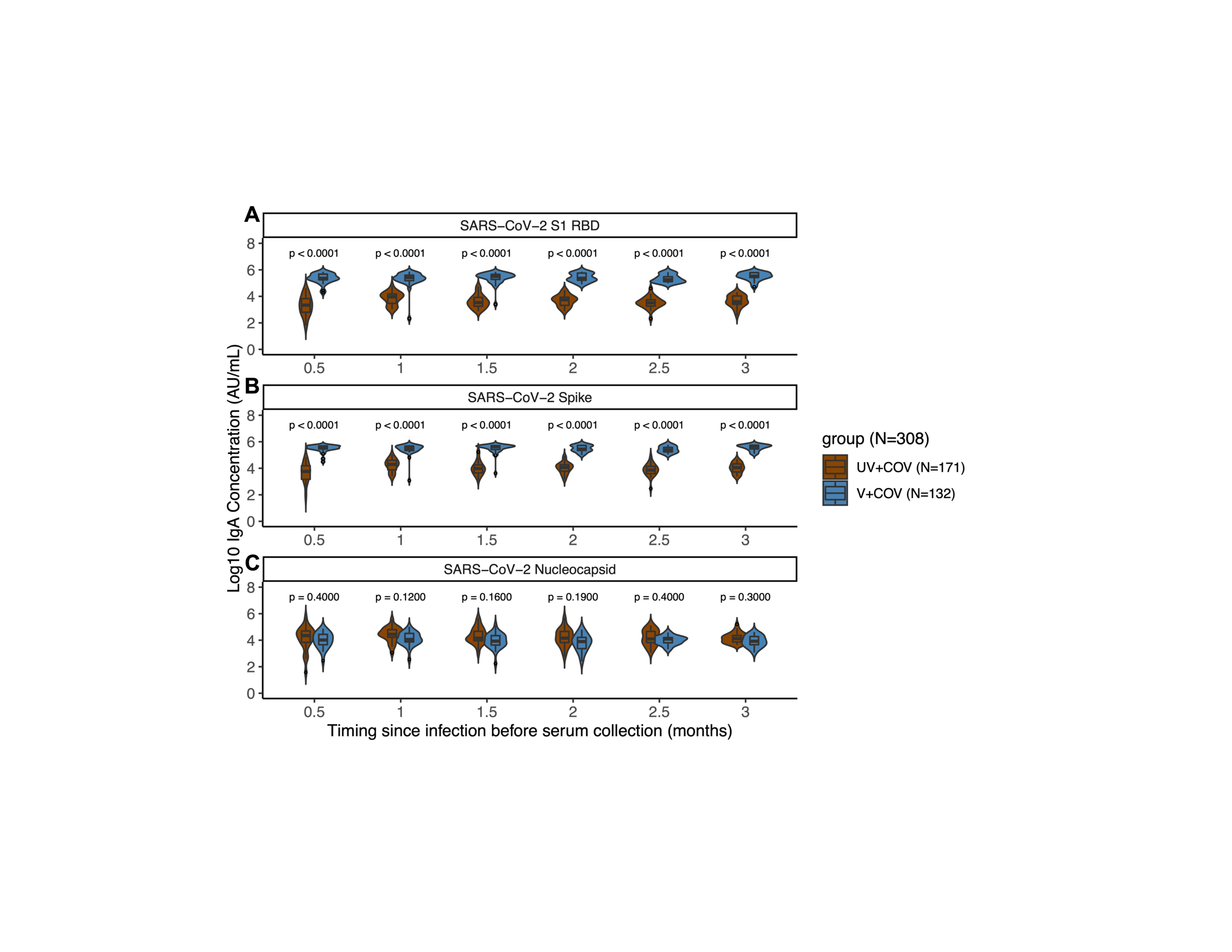


**Supplementary Figure 5. Violin and box plots for comparisons of median IgA levels between vaccinated and unvaccinated pregnant individuals at each timepoint for SARS-CoV-2 RBD (A), S (B), and N (C).** Boxplots describe median concentration and interquartile ranges. Antibody levels in UV+COV (brown) are compared to V+COV (blue) groups. Black dotted lines describe the respective reactivity thresholds in log10 concentration (AU/mL) for SARS-CoV-2 targets. Bonferroni-adjusted Wilcoxon Rank Sum test was used to determine whether the two comparison groups are different at each timepoint, with P-values reported above each comparison. Alpha level for significance was set to P<0.05.

**Supplementary Table 1. Inclusion of study participants by timing group with range. P-value from chi-squared test. UV+COV = unvaccinated prior to infection with SARS-CoV-2. V+COV = vaccinated with two or three doses prior to infection with SARS-CoV-2.**

| Timing group (Days) [Min, Max range of days from PCR positivity to serum collection] | **COVID-19 study population (N=308)** | | **P-value** |
| --- | --- | --- | --- |
|  | **UV+COV (N=171)** | **V+COV (N=137)** | 0.12 |
| 15 (11, 17) | 26 | 26 |  |
| 30 (27, 33) | 26 | 29 |  |
| 45 (41, 49) | 27 | 31 |  |
| 60 (55, 65) | 32 | 20 |  |
| 75 (71, 79) | 26 | 13 |  |
| 90 (86, 95) | 34 | 18 |  |
